# Quantitative proteomic analysis of longissimus dorsi muscle in Chinese and Western pigs using DIA mass spectrometry with PRM validation

**DOI:** 10.1371/journal.pone.0346519

**Published:** 2026-04-30

**Authors:** Hanyu Cao, Xiaojin Li, Fei Xie, Changsheng Jiang, Mengmeng Jin, Ahmed H. Ghonaim, Man Ren, Qianqian Hu, Shenghe Li

**Affiliations:** 1 College of Animal Science, Anhui Science and Technology University, Chuzhou, China; 2 Anhui Province Key Laboratory of Animal Nutritional Regulation and Health, Chuzhou, China; 3 Anhui Engineering Technology Research Center of Pork Quality Control and Enhance, Chuzhou, China; 4 National Key Laboratory of Agricultural Microbiology, College of Veterinary Medicine, Huazhong Agricultural University, Wuhan, China; 5 Desert Research Center, Cairo, Egypt; Central University of Punjab, INDIA

## Abstract

Huoshou black pig (HS) is a well-known indigenous Chinese breed distinguished by superior meat quality compared to Western breeds. To investigate the molecular mechanisms underlying these differences, we performed Data-Independent Acquisition(DIA) proteomic analysis on the longissimus dorsi (LD) muscle from HS and Yorkshire (YY) pigs. We identified 262 differentially expressed proteins (DEPs), including 134 upregulated and 128 downregulated in HS relative to YY. Functional enrichment analysis revealed that these DEPs were significantly involved in small molecule metabolism, oxidoreductase activity, and several key signaling pathways such as the mTOR, AMPK, and PI3K-Akt pathways. Protein -protein interaction network analysis highlighted roles in structural proteins, glycolysis, and ribosome biogenesis. Integrated transcriptomic and proteomic analysis identified five candidate genes (*MGST2*, *PNPO*, *CALD1*, *NCAM1*, *ACSS1*) potentially associated with meat quality traits. Parallel reaction monitoring (PRM) and quantitative PCR (qPCR) validated the consistent differential expression of these genes at both the protein and mRNA levels. These findings provide novel insights into the molecular mechanisms regulating pork quality in indigenous pig breeds.

## Introduction

Pork is one of the primary sources of dietary protein for humans. Intramuscular fat (IMF) content is a key indicator of pork quality, [1] as it directly influences the flavor, tenderness, and juiciness of the meat [2]. Moderately increasing IMF content can significantly enhance these sensory attributes, meeting growing consumer demand for high-quality pork [3]. The level of IMF is determined by a combination of genetic, nutritional, and environmental factors, with key functional genes playing a critical role in IMF deposition in pigs [4,5]. The Huoshou black pig (HS), an indigenous breed originating from Anhui Province, China, is known for its adaptability and superior meat flavor. However, it is challenged by a low lean meat percentage and reduced slaughter efficiency. In contrast, the Yorkshire pig (YY), a widely introduced commercial breed, exhibits high leanness and slaughter efficiency, but inferior meat quality [6,7]. Generally, indigenous Chinese pig breeds show higher IMF content and better meat quality than Western breeds [8], suggesting that HS pigs may harbor key regulatory genes involved in IMF deposition. These genes are likely involved in fat metabolism and muscle development, offering a valuable basis for investigating the molecular mechanisms underlying fat accumulation.

In recent years, advances in transcriptomics and proteomics have greatly contributed to meat quality research, providing powerful tools to uncover the biological mechanisms underlying phenotypic traits [9]. Integrative multi-omics analysis enables a comprehensive understanding of the functional roles and regulatory networks of biomolecules, thereby linking gene expression to protein function more effectively [10]. Several studies have identified differentially expressed genes (DEGs) associated with growth and lipid deposition in Chinese and Western pig breeds using transcriptome sequencing [11]. For example, Wang et al. [12] analyzed the transcriptomes of the longissimus dorsi (LD) muscle in a Chinese local breed (Wuxi pig) and a western lean pig breed (Landrace pig) using RNA sequencing, identifying 682 DEGs related to muscle growth and lipid metabolism. Tan et al. [13] used TMT-tagged proteomic analysis to compare protein expression in glycolytic and oxidative muscles of pigs, identifying 204 differentially expressed proteins (DEPs), involved in muscle fiber type conversion. Similarly, Zhou et al. [14] employed integrated proteomics and lipidomic to study IMF variation in Xidu black pigs, identifying 88 differential proteins and 143 differential lipids between high and low IMF groups. Integrated transcriptome-proteome analysis has emerged as a powerful approach, improving our understanding of complex biological regulatory networks [10,15].

The aim of this study was to investigate the molecular mechanisms underlying meat quality differences between HS and YY pigs through integrated transcriptomic and proteomic analysis. Key genes and proteins associated with IMF deposition were identified at both the mRNA and protein levels. Furthermore, the expression patterns of DEPs were characterized. This study provides novel insights into the genetic and molecular regulation of pork quality traits in Chinese and Western pig breeds, offering a theoretical basis for meat quality improvement through molecular breeding.

## Materials and methods

### Experimental animals and data collection

All experimental pigs—three castrated Huoshou Black (HS1–3) and three castrated Yorkshire (YY1–3)—were supplied by Anhui Anye Agricultural Technology Co., Ltd. and raised under identical housing and nutritional conditions (nutrients levels according to the NRC) with ad libitum feed and water. At 180 days of age, animals were randomly selected and euthanized under protocol No. 2023−218 approved by the Animal Ethics Committee of Anhui Science and Technology University, following the Guidelines for the Care and Use of Laboratory Animals and AVMA recommendations. Unconsciousness was induced by captive-bolt stunning; death was confirmed by absence of corneal reflex, respiration, and heartbeat, followed by exsanguination. LD muscle (between the 3rd and 4th ribs) was immediately excised, snap-frozen in liquid nitrogen, and stored at –80 °C for proteomic analysis.

### Proteome sample preparation

Frozen LD samples were retrieved and placed on ice. The samples were suspended in protein lysis buffer (8 M urea, 1% SDS) containing protease inhibitors and homogenized using a high-throughput tissue grinder (3 cycles, 180 s each). Subsequently, non-contact cryogenic sonication was performed for 30 min. After centrifugation at 16,000 × g and 8°C for 30 min, the protein concentration in the supernatant was determined using the Bicinchoninic Acid (BCA) Protein Assay Kit (Thermo Scientific) following the manufacturer’s protocol.Following quantification, SDS-PAGE was performed for quality control. For digestion, 100 μg of protein was resuspended in 100 mM Triethylammonium bicarbonate buffer (TEAB). The mixture was reduced with Tris(2-carboxyethyl) phosphine (TCEP) at a final concentration of 10 mM at 37°C for 60 min, and then alkylated with iodoacetamide (IAM) at a final concentration of 40 mM at room temperature for 40 min in the dark. After centrifugation at 10,000 × g and 4°C for 20 min, the pellet was collected and resuspended in 100 μL of 100 mM TEAB. Trypsin was added at a 1:50 trypsin-to-protein mass ratio, and the samples were incubated at 37°C overnight.After trypsin digestion, the peptides were dried by vacuum pump. Then, the enzymatically drained peptides were re-solubilized with 0.1% trifluoroacetic acid (TFA), and the peptides were desalted with HLB and dried by vacuum concentrator. Finally, the peptides were quantified using the NANO DROP ONE (Thermo Scientific) by UV absorption value.

### DIA proteomic analysis

Based on peptide quantification results, the peptides were analyzed by an Vanquish Neo coupled with an Orbitrap Astral mass spectrometer (Thermo, USA) at Majorbio Bio-Pharm Technology Co. Ltd. (Shanghai, China). Briefly, the uPAC High Throughptu column (75 μm × 5.5 cm, Thermo, USA) was used with solvent A (water with 2% ACN and 0.1% formic acid) and solvent B (water with 80% ACN and 0.1% formic acid). The chromatography run time was set to 8 minutes. Data-independent acquisition (DIA) data were acquired using an Orbitrap Astral mass spectrometer operated in DIA mode. The mass spectrometry scanning range was 100–1700 m/z.

DIA data was performed using Spectronaut (Version 19) with the Sus scrofa UniProt database (UP000008227, release 20230103, containing 47,945 protein sequences). The parameters are as follows up: The peptide length range was set to 7–52；Enzyme cutting site was trypsin/P；The maximum missed cleavage site was 2；Carbamidomethylation of cysteines as fixed modification, and oxidation of methionines and protein N-terminal acetylation as variable modifications；Protein FDR ≤ 0.01，Peptide FDR ≤ 0.01，Peptide Confidence ≥99%，XIC width≤75 ppm. The protein quantification method was MaxLFQ.

### DEP screening and functional enrichment analysis

Bioinformatic analysis of proteomic data was performed with the Majorbio Cloud platform (https://cloud.majorbio.com). P-values and Fold change (FC) for the proteins between the two groups were calculated using R package “t-test”. The thresholds of fold change (>1.2 or <0.58) and *P-value* <0.05 were used to identify DEPs. Functional annotation of all identified proteins was performed using GO (http://geneontology.org/) and KEGG pathway (http://www.genome.jp/kegg/). DEPs were further used to for GO and KEGG enrichment analysis.

### Protein–Protein interaction (PPI) network analysis

Protein-protein interaction analysis was performed using the String v11.5. Protein-protein interaction (PPI) networks of the DEPs were constructed and visualized using Cytoscape (v3.7.1) software. Network topology was assessed using the CytoHubba plugin to calculate node degree and betweenness centrality. Closely connected modules were identified to further explore the structural characteristics of the network and screen key DEPs associated with meat traits of HS pigs and YY pigs.

### Validation by quantitative real-time PCR (qPCR)

Total RNA was isolated from samples using TRIzol Reagent (Invitrogen) following the manufacturer’s standard protocol. Genomic DNA contamination was eliminated through treatment with DNase I (RNase-free). RNA purity and concentration were determined using a Nanodrop spectrophotometer, with A260/A280 ratios consistently approximating 2.0. RNA integrity was assessed on an Agilent 2100 Bioanalyzer, and all samples exhibited RNA Integrity Number (RIN) values above 8.0.First-strand cDNA was synthesized from 1 μg of total RNA using the PrimeScript™ IV 1st Strand cDNA Synthesis Mix (Takara) with oligo(dT) primers, in a total reaction volume of 10 μL. The thermal profile consisted of 37°C for 15 minutes, followed by 85°C for 5 seconds, and a final hold at 4°C. The resulting cDNA was used immediately in subsequent steps without long-term storage.

Quantitative PCR (qPCR) was performed on a Bio-Rad CFX96 Touch™ system. Each 20 μL reaction contained 50 ng of cDNA, 200 nM of each primer, 3 mM Mg² ⁺ , 200 μM dNTPs, and EZB 2 × SYBR Green qPCR Master Mix (ROX2) (A0001-R2). The thermocycling protocol comprised an initial denaturation at 95°C for 2 minutes; 40 cycles of 95°C for 15 seconds and 60°C for 30 seconds; followed by a melt curve analysis from 65°C to 95°C with increments of 0.5°C every 5 seconds. All reactions were prepared manually using Bio-Rad Hard-Shell® 96-Well PCR Plates (HSP9601) sealed with Microplate Seal Film (MSB1001).Gene-specific primers (listed in Table 1) were designed and manufactured by Shanghai Sangon Biological Engineering Co., Ltd. Primer specificity was verified by melt curve analysis and agarose gel electrophoresis. The annealing temperature was optimized through gradient PCR (55–65°C). Standard curves were constructed from serial dilutions of cDNA, and amplification efficiency was derived from the slope (E = 100% when slope = −3.32).

**Table 1:** qRT-PCR primer information. (*β -actin* is an internal control gene).

Genes	Sequence (5’-to-3’)	Product length	Tm°C
*LMCD1*	F:TTCGAGCCACATTCGTGGAG R:TCAGCAAGCGGCCAATTTTC	109	60.2
*PLIN1*	F:CTGCAGACAAAGTCCTCGGT R:GGGTGTTGGCGGCATATTCA	90	60.5
*FASN*	F:TGGGAAGAGTGTAAGCAGCG R:TGCAGGAACTCGGACATAGC	110	60
*ACSL1*	F:ACAAGTGGAACCACAGGCAA R:TCATCGGAGGAAGGACTGAA	116	58.9
*FABP3*	F:TTGTGACACTGGATGGAGGC R:CCATGGGTGAGTGTCAGGAT	112	59.5
*CSRP3*	F:CAAGGGGATCGGCTATGGAC R:GGCTTTGGGGACTGTTGGAA	90	60.2
*β-actin*	F:AGGCCAACCGTGAGAAGATG R:CATGACAATGCCAGTGGTGC	132	60

All assays demonstrated a linear dynamic range with R² > 0.99.Cq values were automatically determined by the Bio-Rad CFX Maestro™ software using baseline and threshold settings set to default. Technical replicates exhibiting a Cq standard deviation > 0.5 or a coefficient of variation (CV) > 2.5% were discarded. β-actin was selected as the reference gene due to its stable expression across all samples (M value < 0.5, as determined by NormFinder analysis). Relative gene expression was quantified using the 2^(−ΔΔCq) method. Statistical analyses were conducted using GraphPad Prism 9. Data were log-transformed to satisfy normality assumptions and compared using an unpaired t-test or one-way ANOVA followed by Tukey’s post hoc test. Results are expressed as mean ± standard deviation (SD), with a p-value < 0.05 considered statistically significant.

### Parallel reaction monitoring (PRM) validation

To further validate key DEPs, parallel reaction monitoring (PRM)-based targeted mass spectrometry was performed on LD muscle tissues from HS and YY pigs. Proteins were extracted and quantified using the BCA assay and assessed for quality via SDS-PAGE, followed by tryptic digestion and peptide purification Protein identification was conducted in PRM-PASEF acquisition mode using both data-dependent acquisition (DDA) and PRM methods. The primary MS settings included an AGC target of 3 × 10⁶ and maximum ion injection time (Max IT) of 50 ms. Secondary MS used an AGC target of 1 × 10⁵, max IT of 100, and a 2.0 m/z isolation window. The resulting data were searched against a Sus scrofa protein database using MaxQuant. Skyline software was used to develop the PRM method and quantify targeted proteins and peptides.

### Transcriptomic and proteomic integration analysis

Total RNA was extracted from approximately 100 mg of muscle tissue using the TRIzol reagent (Invitrogen, USA) according to the manufacturer’s protocol. RNA integrity was assessed using an Agilent 2100 Bioanalyzer (Agilent Technologies, USA), and only samples with an RIN greater than 8.0 were used for subsequent library construction. Sequencing libraries were prepared with the NEBNext® Ultra™ II RNA Library Prep Kit (NEB, USA) and sequenced on an Illumina NovaSeq 6000 platform (Illumina, USA) to generate 150 bp paired-end reads.Raw sequencing reads were first processed through FastQC (v0.11.9) for quality assessment. Adapters and low-quality bases were trimmed using Trimmomatic (v0.39) with parameters. The high-quality clean reads were then aligned to the Sus scrofa reference genome (Sus_scrofa.Sscrofa11.1) using the STAR aligner (v2.7.10a). Gene expression quantification was performed by counting the number of reads mapped to each gene feature using featureCounts (v2.0.3). A statistical power analysis was conducted using the RNASeqPower R package (online implementation available at https://rodrigo-arcoverde.shinyapps.io/rnaseq_power_alc/) to evaluate the sensitivity of our experimental design.The resulting count matrix was used for differential expression analysis.The statistical power of this experimental design, calculated in RNASeqPower is 0.52.

DEG analysis between the HS and YY groups was performed using the DESeq2 package (v1.34.0) in R. Genes with an adjusted *p-value* (Benjamini-Hochberg FDR) < 0.05 and an absolute |log_2_FC| > 1.2 were considered statistically significant DEGs.The integration analysis was conducted to identify concordant and discordant regulation between the mRNA and protein levels.

Integrated analysis of transcriptomic and proteomic data was conducted to assess the correlation between mRNA and protein expression levels. Pearson correlation analysis was applied and results were visualized using a scatter plot. Furthermore, a nine-quadrant analysis was employed to classify the matched genes into nine distinct categories based on their expression patterns. DEGs and DEPs were compared, and commonly dysregulated molecules were subsequently subjected to functional enrichment analysis. GO and KEGG pathway analyses were performed using the ClusterProfiler and KOBAS tools, respectively. Terms or pathways with an adjusted *p-value* < 0.05 were considered statistically significantly enriched. This integrative approach provided a comprehensive perspective on gene regulatory mechanisms and biological processes associated with IMF deposition.

### Statistical analysis

All statistical analyses were conducted using SPSS 22.0. Data were first tested for normality to determine whether parametric or non-parametric tests were appropriate. For normally distributed data, one-way ANOVA was used. Results are expressed as mean ± SE; and *P* < 0.05 was considered statistically significant.

## Results

### Data quality control

**Fig 1:**
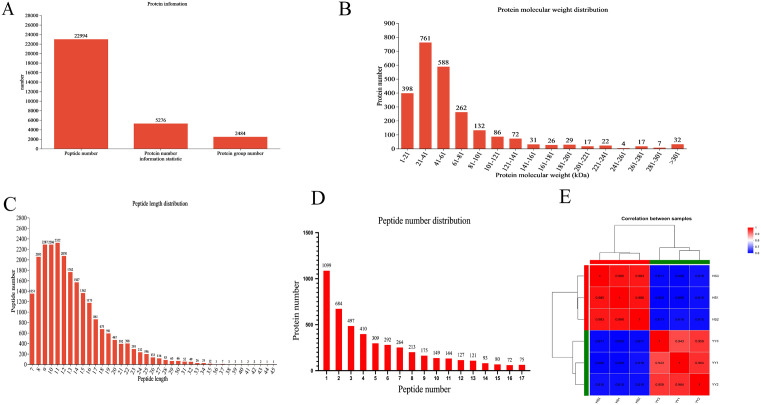
Characterization of proteins from HS and YY longissimus dorsi muscle samples. **(A)** Overview information of identified proteins in this study. **(B)** Distribution of the molecular weights of the identified proteins. **(C)** Distribution of the lengths of the identified peptides.**(D)** Distribution of the numbers of identified proteins containing different numbers of peptides.**(E)** The heatmap of clustering analysis illustrates the correlation between HS and YY samples.

DIA-based proteomics analysis of LD muscle tissues from HS and YY pigs identified a total of 22,994 peptides, 2,484 protein groups, and 5,276 unique proteins (Fig 1A). Fig 1B illustrates that the majority of identified proteins had molecular weights ranging from 21 to 61 kDa, while the distribution of peptide lengths is shown in Fig 1C. As shown in Fig 1D, most proteins were identified by between 1 and 17 peptides, with 1,099 proteins detected by a single peptide. Pearson correlation analysis was performed to assess biological reproducibility between replicates in the two groups. The R^2^ values exceeded 0.99 within groups and 0.80 between groups (Fig 1E), indicating high consistency and reproducibility. Overall, the protein library constructed in this study demonstrates high quality and reliability, meeting the requirements for subsequent quantitative and comparative analyses.

### Differential protein screening

**Fig 2:**
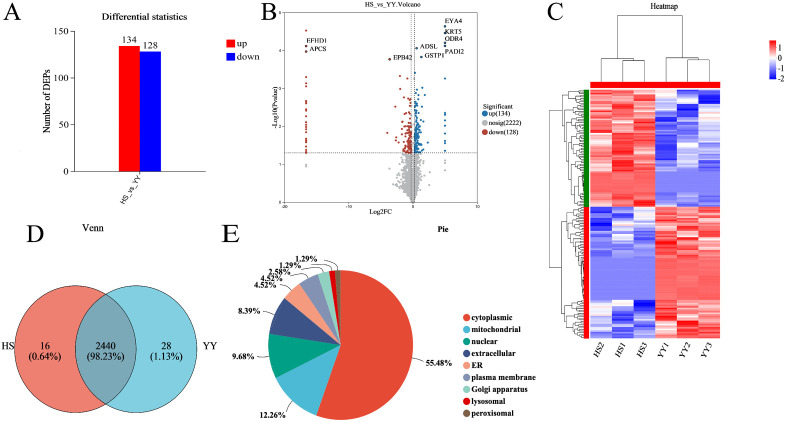
Differentially expressed proteins (DEPs) between the HS and YY pig’s groups. **(A)** bar char plot of proteins in the HS and YY pig’s groups. **(B)** Volcano plot of proteins in the HS and YY pig’s groups. **(C)**Protein Expression Pattern Analysis. The expression values of DEPs, represented as log₂FC, were utilized as input for cluster analysis. Red indicates proteins up-regulated in both HS and YY pigs, while blue signifies proteins down-regulated in both breeds. **(D)**Venn diagram of differential proteins **(E)** Subcellular localization chart of DEPs.

DEPs were analyzed in the LD muscle tissues of HS and YY pigs using MS stats software. A total of 262 DEPs were identified, comprising 134 up-regulated and 128 down-regulated proteins (Fig 2A and 2B). Hierarchical clustering analysis of these DEPs revealed distinct expression patterns that clearly separated the HS and YY pigs (Fig 2C).

A total of 2,440 proteins were found to be commonly expressed in both HS and YY pigs (Fig 2D). Subcellular localization analysis showed that the DEPs were predominantly located in the cytoplasm (55.48%), followed by the mitochondria (12.26%), nucleus (9.68%), and extracellular space (8.39%) (Fig 2E), suggesting their potential roles in diverse cellular processes related to muscle development and metabolism.

### Bioinformatics analysis

**Fig 3:**
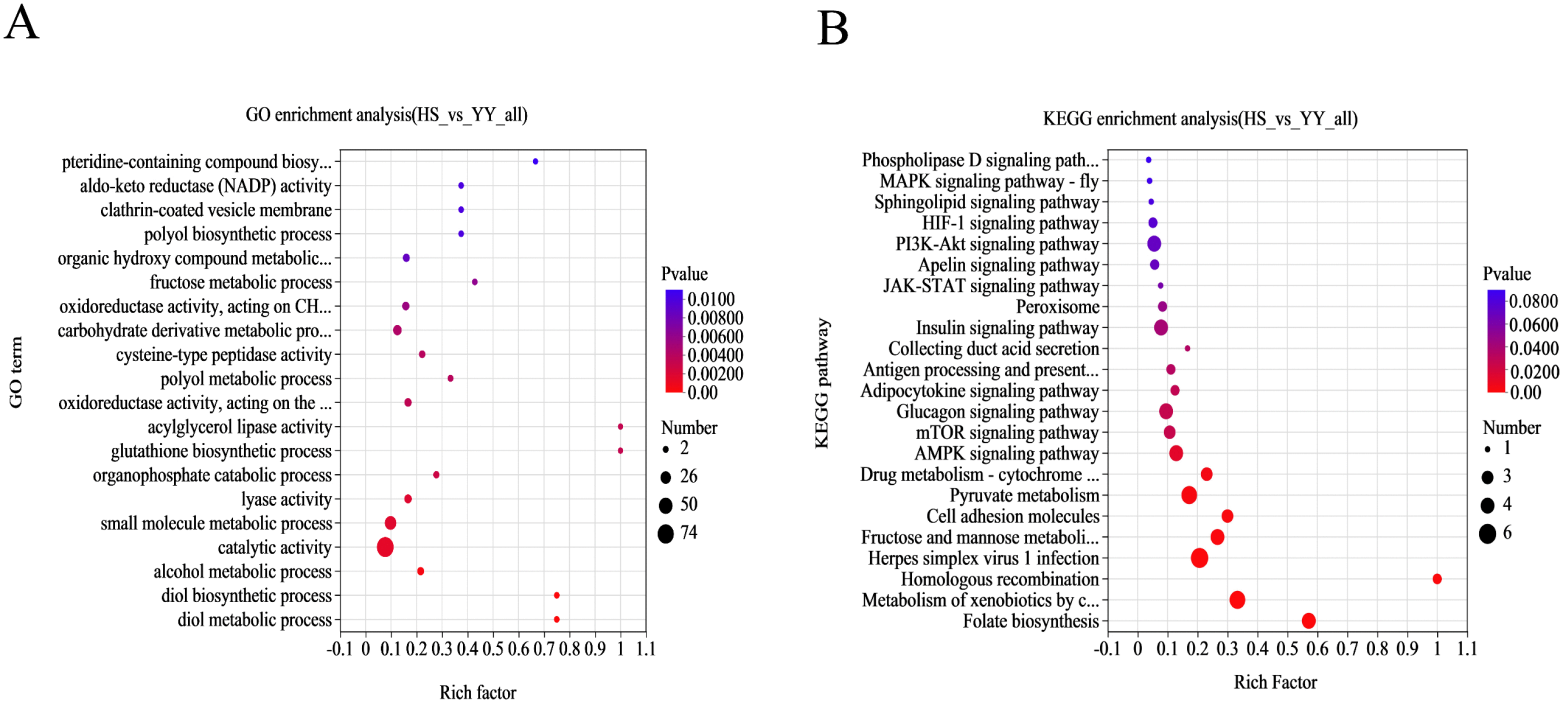
Functional enrichment profiles in HS vs. YY groups. **(A)**GO enrichment analysis of significant DEPs between HS and YY groups. **(B)**KEGG pathway enrichment analysis of significant DEPs between HS and YY groups.

To explore the functional roles of DEPs, Gene Ontology (GO)enrichment analysis was conducted, identifying 92 significantly enriched GO terms (*P* < 0.05), including 65 biological process (BP) terms, 5 cellular component (CC) terms, and 22 molecular function (MF) terms (Fig 3A). Within the BP category, significant enrichment was observed in small molecule metabolic processes, organic cyclic compound metabolic processes, and cellular aromatic compound metabolic processes. For CC terms, the most enriched components were the cytoplasmic vesicle membrane and vesicle membrane. In the MF category, lyase activity, oxidoreductase activity, and catalytic activity were significantly represented. These findings suggest that DEPs are involved in key metabolic and enzymatic processes likely contributing to IMF deposition in HS and YY pigs.

KEGG pathway enrichment analysis (Fig 3B) further revealed 168 pathways associated with DEPs, among which six were significantly enriched (*P* < 0.05), including the AMPK, mTOR, and PI3K-Akt signaling pathways, as well as folate biosynthesis, adipocytokine signaling, and fructose/mannose metabolism. These pathways are known to be involved in energy regulation, lipid metabolism, and adipogenesis, indicating their potential roles in the molecular mechanisms underlying pork quality differences between the two breeds.

### Protein-Protein Interaction (PPI) network analysis

**Fig 4:**
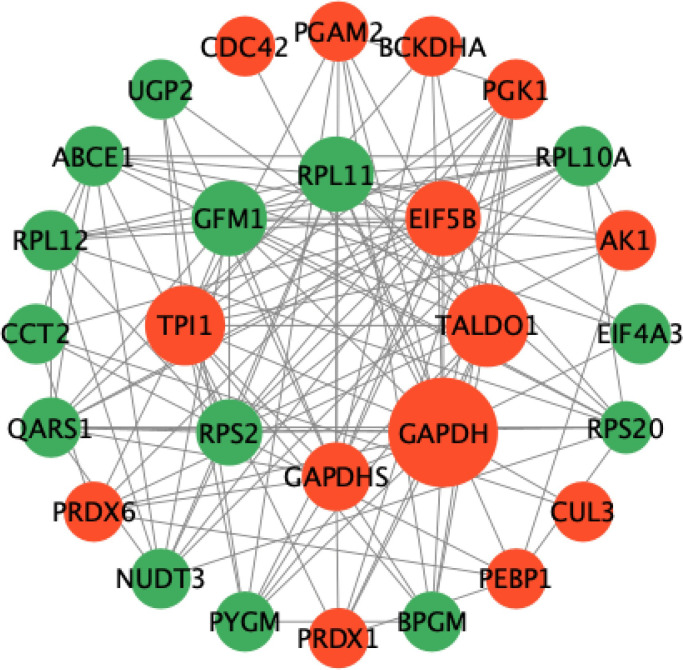
Protein-protein interaction (PPI) network of DEPs. Red nodes represent upregulated proteins, and Green nodes represent downregulated proteins. The size of each node reflects its degree of connectivity, with larger nodes indicating higher interaction degrees.

A protein–protein interaction (PPI) network was constructed using Cytoscape v3.7.1, revealing that the DEPs formed a tightly interconnected network. Functional module analysis indicated that these proteins were primarily involved in the AMPK, mTOR, and PI3K-Akt signaling pathways. Notably, the enrichment of these three pathways in the PPI network was consistent with the KEGG enrichment results, suggesting their central roles in regulating fat deposition within the LD muscle of both YY and HS pigs. The predicted PPI network included 14 upregulated and 14 downregulated proteins (Fig 4). Further network analysis identified several key hub proteins, including GAPDH, GAPDHS, RPS2, TPI1, GFM1, RPL11, EEF1B2, and TALDO1, highlighting their potential importance in mediating molecular interactions that influence muscle metabolism and intramuscular fat accumulation.

### Quantitative RT-PCR validation

**Fig 5:**
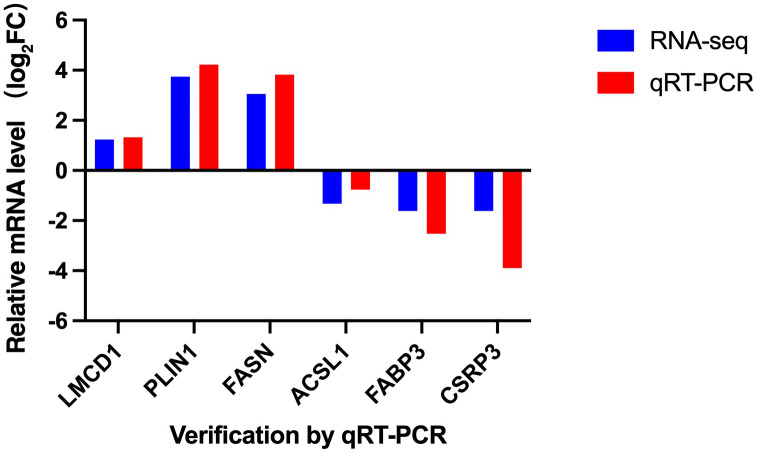
The results of RNA-Seq analysis were verified by qRT-PCR technology.

To verify the reliability of the transcriptome sequencing results, six DEGs were randomly selected for validation by quantitative real-time PCR (qRT-PCR), including three upregulated genes (*LMCD1*, *PLIN1*, and *FASN*) and three downregulated genes (*ACSL1*, *FABP3*, and *CSRP3*). The qRT-PCR results (Fig 5) demonstrated expression trends consistent with those observed transcriptome analysis, confirming the accuracy and reproducibility of the sequencing data.

### PRM validation of proteins

**Fig 6:**
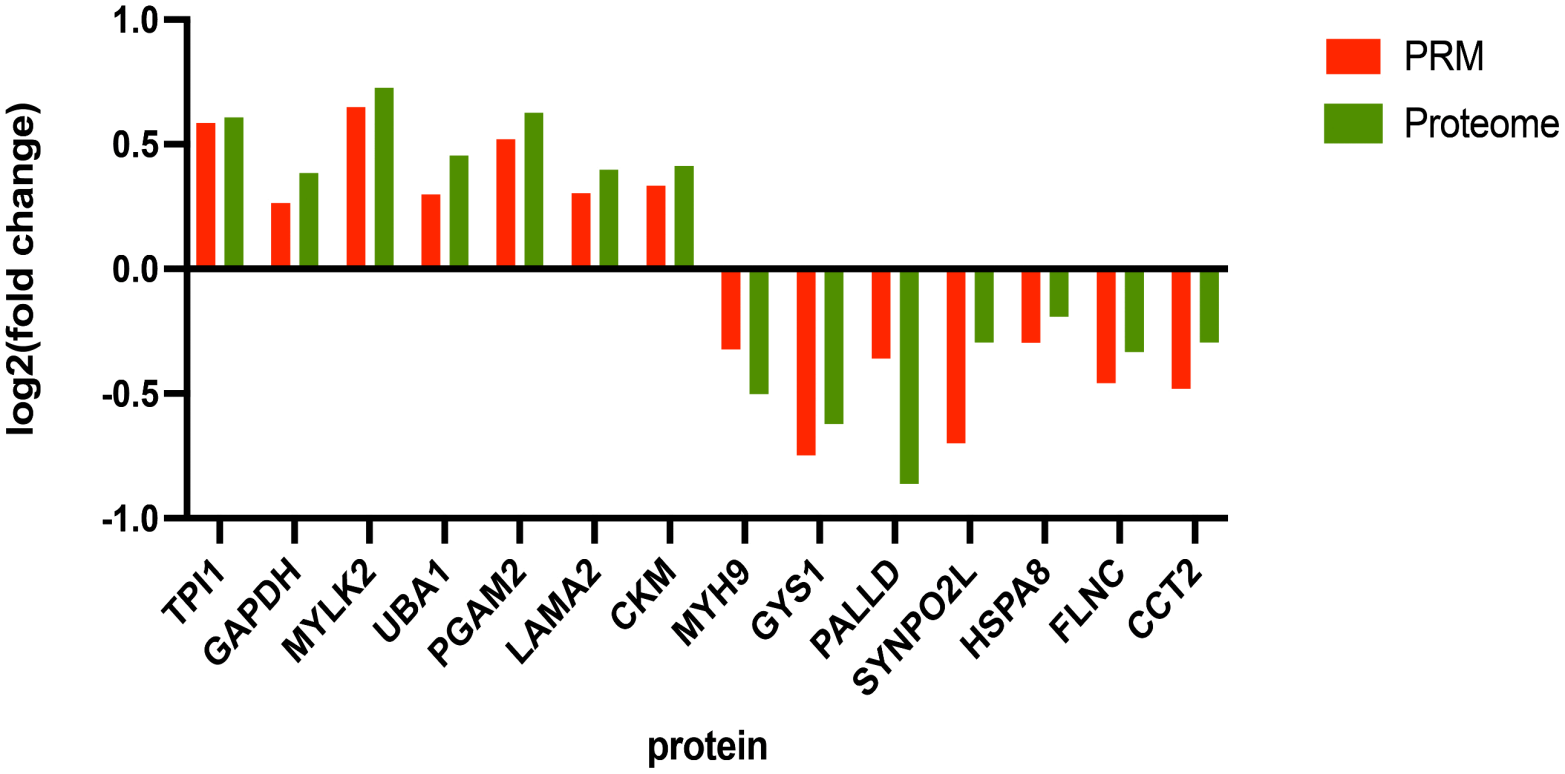
The results of DIA proteomics analysis were verified by using parallel reaction monitoring (PRM) technology.

To confirm the accuracy of the proteomic sequencing results, 14 DEPs were randomly selected for validation using PRM. These proteins included TPI1, GAPDH, GYS1, MYLK2, PALLD, SYNPO2L, UBA1, HSPA8, PGAM2, LAMA2, CCT2, FLNC, CKM, and MYH9. The PRM results (Fig 6) showed expression patterns consistent with those obtained from the DIA-based proteomic analysis, thereby validating the reliability and robustness of the proteomic results.

### Transcriptomic and proteomic integration analysis

**Fig 7:**
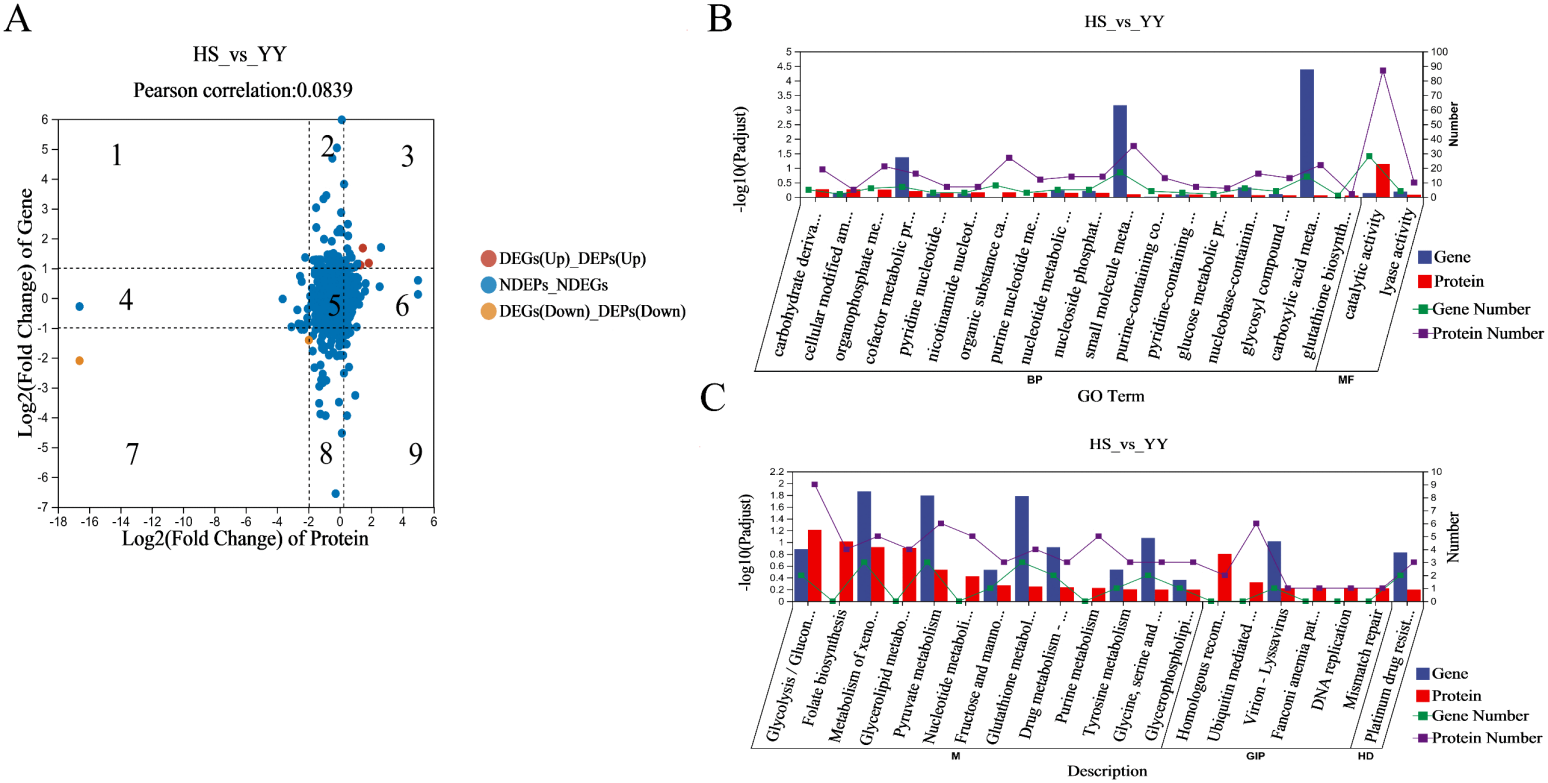
Integrated Analysis of Transcriptome-Proteome Coordination and Functional Enrichment in HS vs. YY Groups (A) Nine-quadrant analysis of transcriptome-proteome coordination in HS vs. YY groups. **(B)** GO enrichment analysis of biological processes in HS vs. YY groups. **(C)** KEGG pathway enrichment analysis in HS vs. YY groups.

To evaluate the complementarity and consistency between transcriptomic and proteomic datasets, we conducted an integrated analysis of DEGs and DEPs. Based on significance thresholds, DEGs and DEPs were categorized into nine quadrants on a correlation plot (Fig 7A). The nine-quadrant analysis revealed a weak but statistically significant correlation between mRNA and protein expression levels (Pearson’s *R* = 0.083, *P* < 0.05). included genes and proteins that were both significantly upregulated, while quadrant 7 contained those that were both significantly downregulated, indicating consistent expression trends across omics levels. The remaining quadrants represented genes or proteins with either no differential expression or inconsistent patterns. Notably, five overlapping genes were identified in quadrants 3 and 7, three significantly upregulated (*MGST2*, *PNPO*, *CALD1*) and two downregulated (*NCAM1*, *ACSS1*), which may play important roles in regulating muscle development and intramuscular fat deposition.

Further functional enrichment analysis using GO and KEGG databases revealed several key biological processes and pathways. Significantly enriched GO terms included cellular amino acid modification, organophosphorus metabolic processes, and macromolecular complex assembly (Fig 7B). The top 20 enriched KEGG pathways featured glycolysis/gluconeogenesis, xenobiotic metabolism, and pyruvate metabolism, underscoring the involvement of energy metabolism and biosynthetic processes in determining meat quality traits (Fig 7C).

## Discussion

Pork quality, an economically important trait, is significantly influenced by IMF content [16]. The quality of pork and the deposition of fat in pig LD muscle are determined by a complex interplay of genes and metabolic pathways [17,18]. To investigate the pronounced differences in meat quality between HS pigs and YY pigs, this study explored the gene expression mechanisms influencing muscle growth and fat deposition. Gene expression, encompassing transcription and protein synthesis, involves RNA as an intermediate and proteins as the functional effectors [19]. While transcriptomic and proteomic approaches each offer valuable insights [20], integrating both enables a more comprehensive understanding of the regulatory mechanisms underpinning meat quality and IMF deposition in the LD muscle of HS and YY pigs.

In this study, DIA -based proteomics identified 262 DEPs between HS and YY pigs, including 134 upregulated and 128 downregulated proteins. Notably, MYBPC1 and CKM were significantly upregulated in HS pigs. MYBPC1 (myosin-binding protein C, slow-twitch fiber type) is crucial for muscle contraction and relaxation [21]. CKM (creatine kinase M-type), a muscle-specific enzyme, plays an essential role in energy metabolism and muscle contraction and growth [22]. Previous studies have shown a positive correlation between CKM expression and muscle growth rate and fiber development in pig breeds such as the Two-Guang Small Flower pig [23]. In contrast, the most significantly downregulated proteins in HS pigs were FLNC and RPS6KA3. FLNC (Filamin C), a key actin-binding protein in striated muscle cells [24], maintains and repairs myofibrils under mechanical stress, and exhibits dynamic responses in muscle cells [25]. Its lower expression in Diannan small-ear pigs compared to Landrace pigs has been linked to reduced muscle repair capacity [26]. RPS6KA3, a serine/threonine kinase, is main participation involved in the cell growth and differentiation [27]. Jiao et al. [28] reported its expression to be lower in Chinese pig breeds relative to Western pig breeds. The observed upregulation of MYBPC1 and CKM, alongside downregulation of FLNC and RPS6KA3 in HS pigs, suggests breed-specific gene expression patterns that likely impact muscle growth, IMF accumulation, and overall pork quality.

GO enrichment analysis revealed that IMF deposition and muscle growth differences between HS and YY pigs were closely associated with small molecule metabolic processes and organic cyclic compound metabolism. KEGG pathway and PPI network analyses showed that DEPs in HS pigs were significantly enriched in the AMPK, mTOR, and PI3K-Akt signaling pathways, relative to YY pigs. Adenosine monophosphate-activated protein kinase (AMPK) is a key regulator of cellular energy metabolism, often referred to as the cellular energy sensor, and plays numerous biological roles [29,30]. AMPK is the core gene of the AMPK signaling pathway. Upon activation, AMPK promotes lipid oxidation and glucose uptake, while inhibiting lipid synthesis and reducing IMF content [31]. Therefore, AMPK activity is negatively correlated with IMF deposition. In this study, AMPK was highly expressed in the LD muscle of the YY pig, resulting in lower IMF content. This finding aligns with previous research [32]. During skeletal muscle development, muscle fiber hypertrophy is crucial for increasing skeletal muscle mass. The mammalian target of rapamycin (mTOR) is the most crucial signaling pathway involved in the process of muscle fiber hypertrophy [33]. Torrazza et al. [34]found that Leu can enhance the rate of muscle protein synthesis by increasing the phosphorylation levels of mTOR and 4EBP1 proteins, thereby promoting growth and development in pigs. The phosphatidylinositol 3-kinase/protein kinase B (PI3K/Akt) signaling pathway mainly participates in regulating the proliferation, growth, differentiation and apoptosis of skeletal muscle cells [35]. PI3K-Akt contributes to muscle protein synthesis by inhibiting GSK-3β and promotes muscle growth through regulation of cellular metabolism [36]. Furthermore, in terms of fat deposition, PI3K-Akt signaling pathway supports fat deposition by modulating. lipogenesis-related transcription factors such as PPARγ [37]. The PPI network analysis identified GAPDH, GAPDHS, RPS2, TPI1, GFM1, RPL11, EEF1B2, and TALDO1 as key hub proteins related to fat metabolism and muscle development. TPI1 (Triosephosphate isomerase 1), a glycolytic enzyme, is essential for maintaining cellular energy supply during muscle contraction [38]. GAPDH (Glyceraldehyde-3-phosphate dehydrogenase) also plays a central role in glycolysis [39]. GAPDH modulates mTOR signaling, thereby influencing myoblast differentiation and myotube formation [40]. RPS2, a component of the 40S ribosomal subunit, is critical for mRNA decoding and protein synthesis [41]. In our study, TPI1 and GAPDH expression were significantly upregulated in HS pigs than in YY pigs, indicating enhanced glycolytic capacity for ATP production and endurance in LD muscle, whereas RPS2 was downregulated, potentially reflecting slower protein synthesis rates. Together, these proteins interact to regulate muscle growth development and fat storage, which may account for the higher IMF levels observed in HS pigs.

This study found a low correlation between mRNA and protein expression levels (Pearson’s R = 0.083). This phenomenon is widespread in systems biology research, indicating that gene expression is finely regulated at multiple levels post-transcription [42]. First, protein degradation and turnover are key factors leading to the discrepancy, as differences in protein half-lives may cause protein abundance to fluctuate independently of mRNA levels [43]. Second, translational regulation limits the efficiency of converting transcripts into proteins [44]. Additionally, post-translational modifications (PTMs) not only affect protein function but also influence detected abundance by altering protein stability [45]. Our findings underscore that mRNA abundance is a poor proxy for protein expression in this context, reinforcing the importance of integrated proteo-transcriptomic profiling to provide a more accurate representation of the functional landscape of pork quality. Integrated transcriptomic and proteomic analysis identified five genes, *MGST2*, *PNPO*, *CALD1*, *NCAM1*, and *ACSS1*, that were differentially expressed at both mRNA and protein levels between HS and YY pigs. Among them, *MGST2, PNPO*, and *CALD1* were significantly upregulated, while *NCAM1* and *ACSS1*) were downregulated in HS pigs. *MGST2* is associated with fatty acid metabolism [46], and has been linked to variation in fatty acid composition, particularly C16:0 and C16:1(n-7), in pig longissimus muscle, indicating its potential role in fatty acid synthesis, degradation, or transport [47]. *CALD1* encodes an action-binding protein involved in cytoskeletal remodeling and muscle contraction regulation. *ACSS1* regulates intracellular acetyl-CoA metabolism, contributing to lipid droplet formation and fat storage [48,49]. Collectively, these five genes represent promising candidates for regulating IMF deposition and muscle development and could serve as potential targets for genetic selection aimed at improving pork quality and growth traits. However, further functional studies are necessary to validate their biological roles and their applicability in breeding programs.

We acknowledge that the statistical power of our RNA-Seq design (0.52) is a limitation of this study. A power of this magnitude suggests a non-negligible risk of Type II errors, meaning that some biologically relevant genes might not have reached statistical significance in our analysis. Consequently, the findings presented here should be interpreted as exploratory rather than conclusive. However, the integration of proteomic data serves as a critical filter, identifying robust molecular signatures that persist across different levels of gene expression. Future studies with larger cohorts are required to validate these candidates and capture the full spectrum of the molecular landscape.

## Conclusions

In summary, we conducted a DIA-based proteomics analysis of LD muscle from HS and YY pigs, identifying 262 DEPs (134 upregulated and 128 downregulated) associated with differences in IMF content. These findings improve our understanding of the molecular basis of IMF differences at the protein level. Furthermore, integrated transcriptomic and proteomic analyses revealed five key genes related to fat deposition and muscle development, three upregulated (*MGST2, PNPO, CALD1*) and two downregulated (*NCAM1, ACSS1*). Among these, *CALD1, NCAM1*, and *PNPO* are primarily involved in muscle growth, while *ACSS1* and *MGST2* are closely associated with lipid deposition. This study provides novel insights into the molecular mechanisms governing adipose accumulation and muscle development in porcine LD muscle from a proteomic perspective. It offers a theoretical framework for understanding the biological processes of muscle formation and lipid metabolism and lays a valuable foundation for future research aimed at improving pork quality through genetic and molecular approaches.
